# Initial COVID-19 Transmissibility and Three Gaseous Air Pollutants (NO_2_, SO_2_, and CO): A Nationwide Ecological Study in China

**DOI:** 10.3389/fmed.2020.575839

**Published:** 2020-09-24

**Authors:** Jinjun Ran, Shi Zhao, Lefei Han, Zhihang Peng, Maggie H. Wang, Yulan Qiu, Daihai He

**Affiliations:** ^1^Li Ka Shing Faculty of Medicine, School of Public Health, The University of Hong Kong, Hong Kong, China; ^2^School of Public Health, Shanghai Jiao Tong University School of Medicine, Shanghai, China; ^3^Jockey Club School of Public Health and Primary Care, Chinese University of Hong Kong, Hong Kong, China; ^4^Shenzhen Research Institute, Chinese University of Hong Kong, Shenzhen, China; ^5^School of Nursing, The Hong Kong Polytechnic University, Hong Kong, China; ^6^School of Public Health, Nanjing Medical University, Nanjing, China; ^7^Department of Applied Mathematics, Hong Kong Polytechnic University, Hong Kong, China

**Keywords:** gaseous air pollutant, COVID-19, transmissibility, ecological study, China

## Abstract

In this study, we conducted an ecological study to examine their effects in the early phase of the pandemic (from December 2019 to February 2020) in China. We found that the associations between the average concentrations of NO_2_, SO_2_, and CO and the COVID-19 transmissibility are not statistically clear.

## Introduction

The ongoing Coronavirus 2019–20 pandemic has caused a huge impact on global public health. Many studies showed that a high level of air pollutant concentrations increases the risk of pneumonia infection and deaths. The pollutant concentrations were significant for severe acute respiratory infection (1). Nitrogen dioxide (NO_2_) exposure increases the occurrence, severity, hospitalization, and 30-day mortality, especially in cold months (2). Long-term exposure to higher levels of NO_2_ increases the increased hospitalization for community-acquired pneumonia (CAP) in older adults (3). Short-term exposure to higher levels of NO_2_ and carbon monoxide (CO) increases pneumonia-related hospitalization, emergency department visits, and outpatient visits in adults (4, 5). The concentrations of NO_2_, sulfur dioxide (SO_2_), and CO were positively associated with the upper respiratory tract infection and CAP (6). Becker and Soukop reported a non-linear dose-response effect between respiratory syncytial virus (RSV) internalization by airway epithelial cells and the level of NO_2_ (positive association at a low-level 0.5 ppm of NO_2_ and a negative association at a high-level 1.5 ppm) (7). Both the release of infectious virus 48-h post-exposure and virus-induced cytokine production were reduced at a high level of NO_2_.

It is of significance to study the impact of gaseous air pollutants, especially NO_2_, SO_2_, and CO on the transmission of coronavirus diseases 2019 (COVID-19) in China. Many studies have been done on the impact of meteorological factors and air pollutants on the spread and COVID-19 and the patient outcome, for example, focusing in China (8–11), as well as other studies focusing in Italy, Spain, and the United States. However, some of these studies adopted a chronic disease approach, using either the linear regression model or time series model. However, COVID-19 is a highly transmissible infectious disease. We cannot merely adopt a statistical model for chronic disease to gain insights on the effects on the transmissibility (12). For instance, the serial correlation in daily new cases should be removed in a time series model (9). Riccò et al. (13) pointed out several other issues. For example, the lockdown of cities not only reduced the transmission of COVID-19 but also reduced the level of air pollution. Thus, the correlation between the level of certain pollutants and the transmission rate over time does not mean causation. The correlation between the transmission and meteorological conditions and air pollution could be non-linear (11).

## Methods

We collected daily confirmed COVID-19 cases from 303 cities in China from the Chinese provincial health agencies and China National Health Commission. We calculated the basic reproductive number, *R*_0_, which is a unit-free measure of infectivity of a virus and is commonly used in infectious disease epidemiology. For the estimation of *R*_0_, we first estimated the exponential (or intrinsic) growth rate, denoted by *r*, of the epidemic curve over the a 16-day period starting from the confirmation of the first case in each city (14–16). The number of cases at the *t*-th day, *C*_*t*_, is modeled as *C*_*t*_ = *C*_0_exp(*rt*), where *C*_0_ denoted the number of seed cases at the start of the outbreak. Using the formula *R*_0_ = 1/*M*(–*r*) that is derived from the Lotka-Euler equation (17), we substitute *r* into the moment generation function, *M*(·), of the probability distribution of the COVID-19 serial interval. The distribution of serial interval is approximated by a Gamma distribution with mean at 5.5 days and standard deviation at 3.3 days (17–20). This analytical approach is also used in previous COVID-19 studies as well as in other infectious diseases (20–23).

We obtained air pollutant data in 1,642 observation stations from the China National Environmental Center and obtained meteorological data from the National Meteorological Data Center. We computed a raster for each pollutant by the kriging interpolation based on the averaged values across the period December 10, 2019, to February 29, 2020. Then we interpolated the level of each pollutant for each city. We adopted generalized (i) univariable, and (ii) multivariable linear regression, using *R*_0_ as the response and air pollutant as the factor, adjusted by temperature and relative humidity. In addition, we employ the spline regression for sensitivity analysis.

## Results

**Figure 1:**
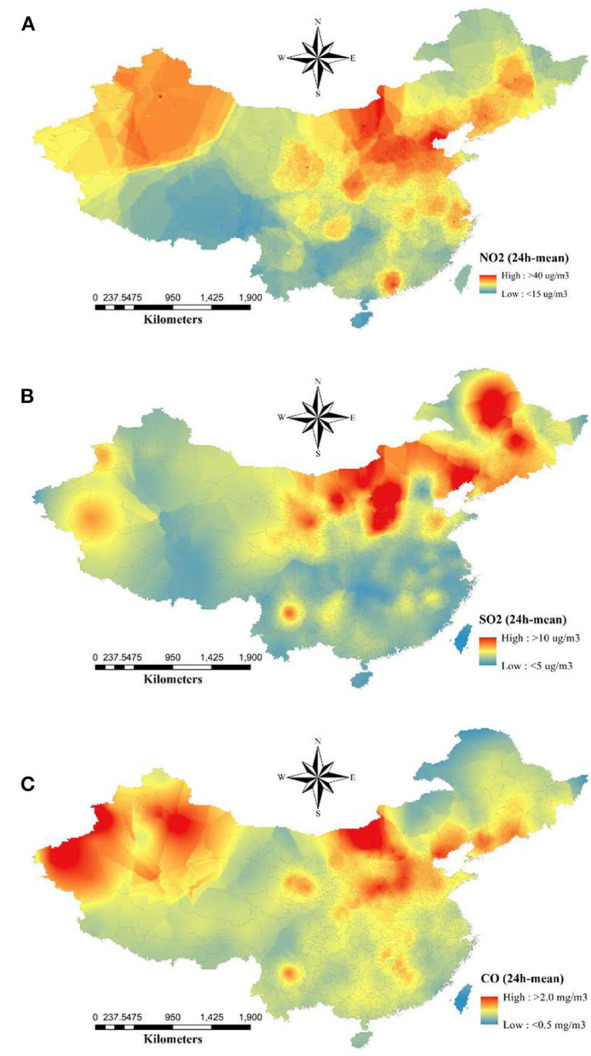
Spatial distribution of average concentrations of ambient **(A)** NO_2_, **(B)** SO_2_, and **(C)** CO from December 10, 2019 to February 29, 2020. The raster maps were converted by the kriging interpolation after averaging their concentrations.

The descriptive statistics of the basic productive numbers, three pollutants, temperature, and relative humidity for 154 cities are given in Table 1. The maximum *R*_0_ = 2.5 was detected in Wuhan City. The average concentrations of NO_2_, SO_2_, and CO were 31.0, 11.9 μg/m^3^, and 1.0 mg/m^3^, respectively. The spatial distributions of NO_2_, SO_2_, and CO are shown in Figure 1. Pearson and Spearman's ranked correlation coefficients between the level of pollutants and the basic reproductive number are given in Table 2. We observed no significant correlation.

**Table 1:** Descriptive statistics of the basic reproductive numbers, three gaseous pollutants, temperature, and relative humidity across 154 Chinese cities.

	**Mean**	**SD**	**Min**	**25th**	**Median**	**75th**	**Max**	**IQR**
**COVID-19 transmissibility**								
*R*_0_	1.4	0.3	1.0	1.1	1.3	1.5	2.5	0.4
**Gaseous pollutants**								
NO_2_ (μg/m^3^)	31.0	7.8	13.1	25.7	31.1	36.1	49.3	10.4
SO_2_ (μg/m^3^)	11.9	6.0	5.3	7.8	9.7	14.3	42.2	6.5
CO (mg/m^3^)	1.0	0.2	0.5	0.8	0.9	1.1	1.7	0.3
**Weather conditions**								
Temperature (°C)	4.3	8.3	−22.5	0.4	6.2	9.3	19.1	8.9
RH (%)	73.9	9.2	42.2	67.9	75.6	81.0	89.0	13.1

*R_0_, basic reproductive number; NO_2_, nitrogen dioxide; SO_2_, sulfur dioxide; CO, carbon monoxide; RH, relative humidity; SD, standard deviation; IQR, interquartile range*.

**Table 2:** Correlation coefficients of COVID-19 *R*_0_s with the three gaseous pollutants across 154 Chinese cities.

	**Pearson's correlation**		**Spearman's ranked correlation**	
	**Estimate**	***p*-value**	**Estimate**	***p*-value**
NO_2_	0.01	0.896	0.02	0.866
SO_2_	−0.09	0.264	−0.12	0.143
CO	−0.01	0.855	0.04	0.661

*NO_2_, nitrogen dioxide; SO_2_, sulfur dioxide; CO, carbon monoxide*.

**Figure 2:**
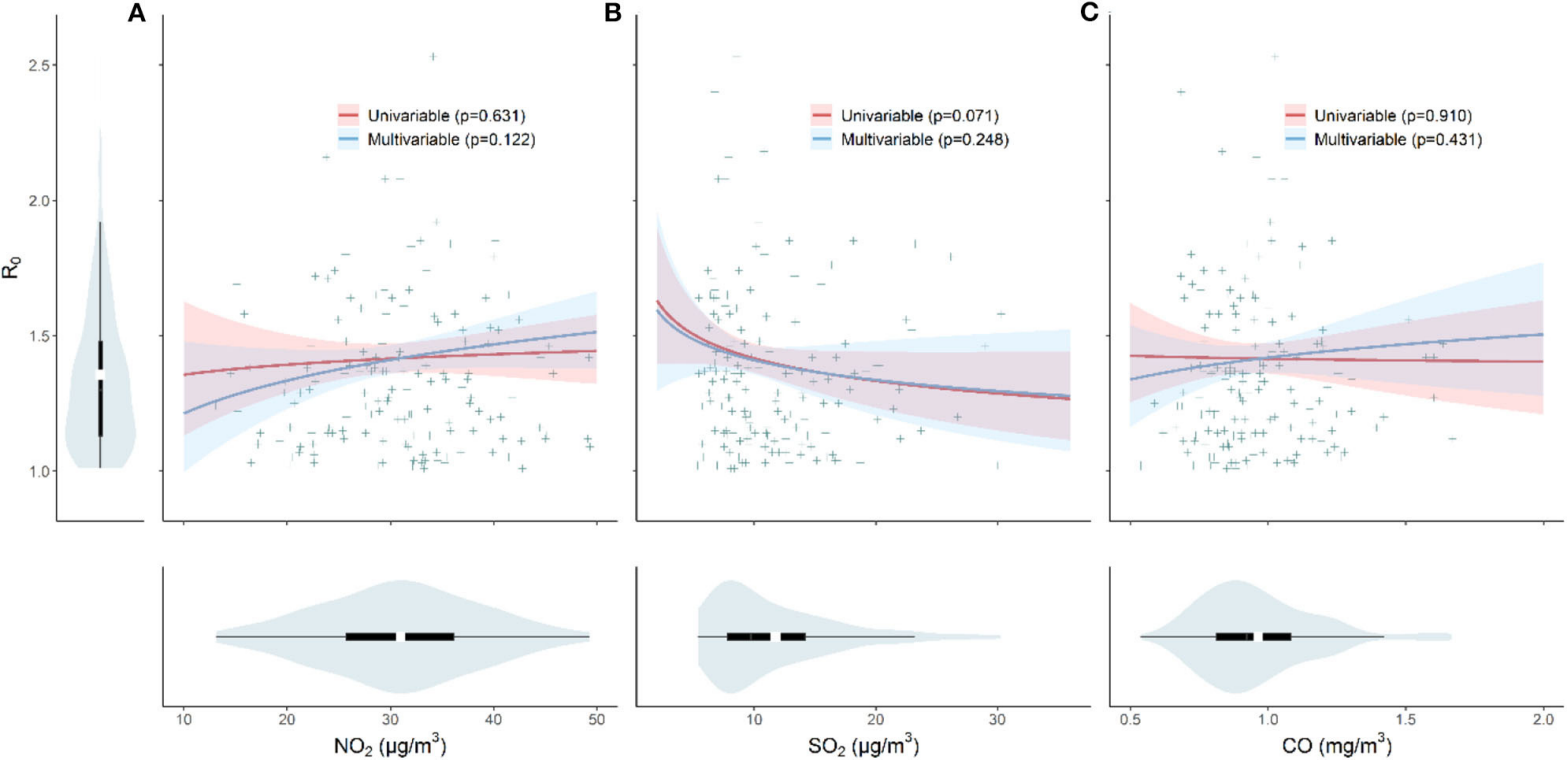
The effects of pollutant **(A)** NO_2_, **(B)** SO_2_, and **(C)** CO concentrations and the initial transmissibility of COVID-19. Violin plots shows the distributions of *R*_0_, NO_2_, SO_2_, and CO, respectively. The red line indicates the result from the univariable model, and the blue line represents result from the multivariable model (adjusted for temperature and relative humidity). The *p*-values are given in the legend.

In Figure 2, using either univariable or multivariable linear regression, we found no clear effects of NO_2_, SO_2_, and CO on the initial transmissibility of COVID-19 across Chinese cities. We performed sensitive tests by using spline regression (three degrees of freedom) and restricting the cities outside Hubei province only and found no significant association.

## Discussion

The merit of this work is that we focus on the transmissibility in the initial phase of the pandemics in each city, which may reflect the intrinsic feature of the local outbreak to some extent. However, several limitations should be noted. Population density is a potential confounder since it may be positively related to gaseous pollutant concentrations and may catalyze the spread of COVID-19 (24, 25). Besides observed associations are not of statistical significance between gaseous air pollutants and the COVID-19 transmission, we may still overestimate the true effects because the unadjusted confounder, population density, would positively bias the associations (26). Population flow from Wuhan City may be another potential confounding factor since it may result in disentangled imported or local cases for other Chinese cities. An emerging study found that correlations between gaseous pollutants and *R*_0_ varied in provinces with different population flow (27). However, the effect is likely uniform across cities, and the lockdown of Wuhan mitigated the COVID-19 exportation to other cities effectively. Previously works found the importation was reasonable uniform except for very few cities. This approach has been used previously by other teams and our team (28–30). We avoid using a time series model over a more extended period since it may suffer more issues discussed and demonstrated in a recent study (13). We argue that if there were indeed an “evident” statistical association, our approach should pick it up. At least, we avoid picking up a spurious association, for example, the entangled effects between pollution reduction (if any) and city lockdown. Although the results are not of statistical significance, our method is straightforward on one hand, and we nevertheless consider the possibility of the non-linearity of association on the other hand.

In this large-scale ecological study, we find no significant association between the three gaseous air pollutants (NO_2_, SO_2_, and CO) and the initial transmissibility of COVID-19 in Chinese cities. Since we focus on the initial 16 days after the confirmation of the first case, the lockdown of cities should not have reduced the pollutant level in each city, as normally it will take time for pollutants to drop. Our averaged concentration should be a fair indicator of the level of air quality in each city.

Other factors for the insignificant results include that the outbreak period covered the spring festival when factories were closed. And the closure of factories was extended by the government. Many cities adopted different levels of lockdown, and emission of vehicles were reduced. Thus, the concentration of air pollutants may be low in general. Strict and effective mitigation measures stopped the transmission timely.

## Data Availability Statement

All datasets presented in this study are included in the article/Supplementary Material.

## Author Contributions

JR and SZ conceived the study and carried out the analysis. LH collected the data. JR, SZ, and DH drafted the letter and discussed the results. All authors critically read and revised the letter and gave final approval for publication.

## Conflict of Interest

The authors declare that the research was conducted in the absence of any commercial or financial relationships that could be construed as a potential conflict of interest.
