# Supplementary material for: Initial COVID-19 Transmissibility and Three Gaseous Air Pollutants (NO2, SO2, and CO): A Nationwide Ecological Study in China
Source: Front Med (Lausanne). 2020 Sep 24;7:575839. doi: 10.3389/fmed.2020.575839 (PMC7541936; doi:10.3389/fmed.2020.575839)
Supplement: Supplementary file 1 [file Data_Sheet_1.PDF]

Initial COVID-19 Transmissibility and Three Gaseous Air Pollutants (NO2, SO2 and CO): A Nationwide Ecological Study in China

Supplementary Materials

1. Relationships of the R_0s with NO_2, SO_2, and CO concentrations in the cities outside Hubei

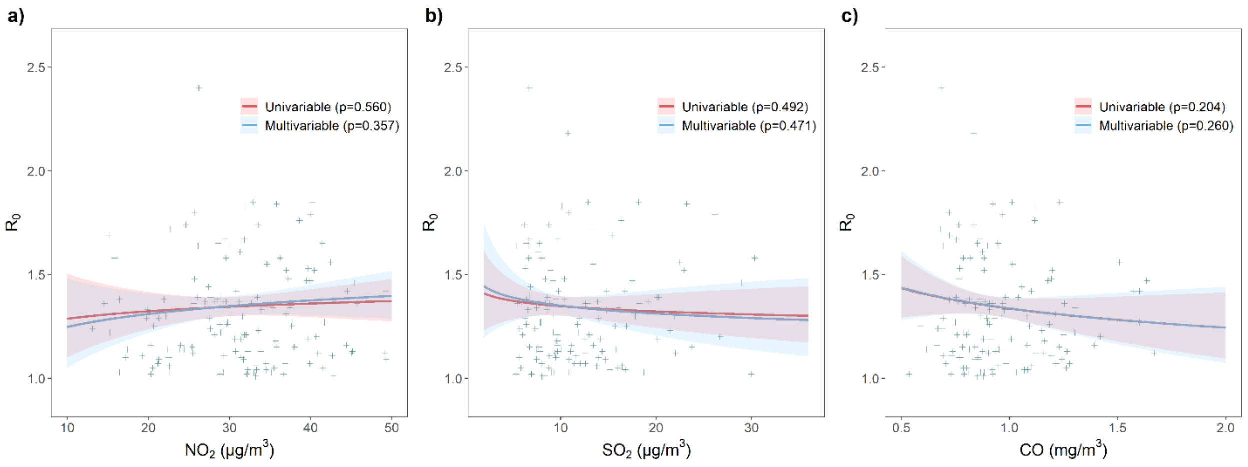

Supplementary Figure 1. The relationships of the COVID-19 R_0s with NO_2, SO_2, and CO concentrations in the Chinese cities outside Hubei. The red and blue lines indicate the relationship in the univariable and multivariable regressions, respectively. The *p*-value ≥ 0.05 means the null-hypothesis is not rejected. Panels A, B, C show the relationships with NO_2, SO_2, and CO concentrations, respectively.

2. Relationships of R_0 with the three gaseous pollutants by spline regression

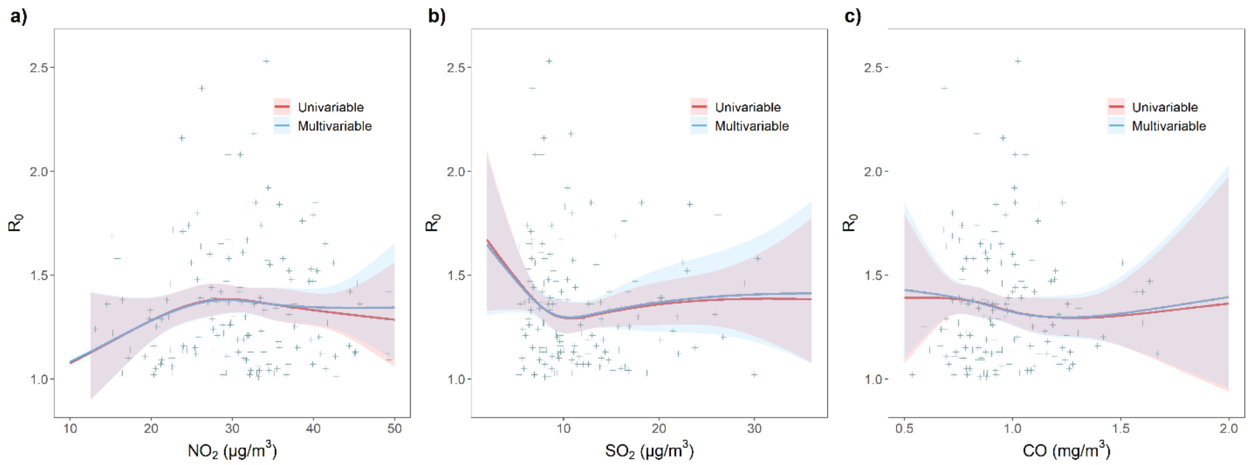

Supplementary Figure 2. Estimated relationships between the COVID-19 R_0s and the three gaseous pollutants conducted by the natural spline regression with three degrees of freedom. Panel A, B, C show the relationships with NO_2, SO_2, and CO concentrations, respectively.
